# Addressing Behavioral and Psychiatric Symptoms in Dystonia: A Case Report of Fahr Disease

**DOI:** 10.1002/ccr3.72654

**Published:** 2026-05-10

**Authors:** Tara Khoeini, Mohammad Javad Sinaeefar

**Affiliations:** ^1^ Department of Neurology, Firoozgar Hospital, School of Medicine Iran University of Medical Sciences Tehran Iran

**Keywords:** basal ganglia calcification, fahr's disease, fahr's syndrome, movement disorders

## Abstract

Abnormal calcium deposits in areas of the brain that control movement, including basal ganglia and cerebellum, are the hallmark of Fahr's syndrome. This report highlights the importance of clinicians being vigilant regarding behavioral and neuropsychiatric symptoms when evaluating cases of dystonia. These important clues indicate underlying inherited and neurodegenerative disorders.

## 1 Introduction

Fahr's disease, named after German neurologist Karl Theodor Fahr, was first reported in 1930. This rare neurological condition is characterized by abnormal calcium deposits in the basal ganglia and subcortical white matter. It is commonly inherited in about 60% of diagnoses in an autosomal dominant manner [1].

The symptoms of Fahr disease, also known as bilateral striatopallidodentate calcinosis, dysarthria, seizure, involuntary movements, dementia, and vision impairment [2, 3]. This syndrome can present differently in each patient, and there is no clear correlation between the brain lesion and the clinical symptoms [1].

Calcifications in the caudate nucleus and putamen (and perivascular regions) damage nigrostriatal projections from the substantia nigra and interfere with dopaminergic terminals in the striatum. This reduces dopaminergic transmission in the nigrostriatal pathway, contributing to parkinsonian signs and altered motor/behavioral regulation. Calcifications in the caudate nucleus and putamen (and perivascular regions) damage nigrostriatal projections from the substantia nigra and interfere with dopaminergic terminals in the striatum. This reduces dopaminergic transmission in the nigrostriatal pathway, contributing to parkinsonian signs and altered motor/behavioral regulation. Fahr‐related small‐vessel disease and chronic hypoperfusion can cause selective vulnerability and degeneration of dopaminergic neurons, further diminishing striatal dopamine availability [4, 5].

## 2 Case History/Examination

A 29‐year‐old right‐handed, single, and unemployed woman was referred to Firoozgar Hospital, Tehran, Iran, due to behavioral changes and abnormal posturing in her hands in January 2024. Symptoms began 5 years ago when the patient slowly developed behavioral changes in the form of aggression and withdrawal behaviors and mood disorders, as well as abnormal movements and postures in the left hand and dysarthria. Subsequently, 3 years later, the patient also experienced a gait disorder that has been worsening progressively over this period. There was no history of seizure, memory loss, or visuospatial disorder, as well as no symptoms of hallucinations, delirium, or catatonia.

In her personal history, she had normal motor milestones, although her primary education was restricted because of difficulties in learning, leading her to discontinue schooling at the age of 11. In the family history, the patient's parents were first cousins. There were no similar symptoms in other family members. Psychological examination showed a disorganized and slightly disheveled appearance with psychomotor slowness; she had good visual and verbal communication. Consciousness, orientation to time, place, and persons, and also the state of memory, were normal.

In the subsequent examinations, she had a Mini‐Mental State Examination (MMSE) score of 22/30. In the neurological examination, she had slurred speech, dystonic posture in her left hand, mild bradykinesia and a masked face and also dystonic posture in the left foot, which was exaggerated while standing and walking. Kayser–Fleischer (KF) ring was not detected. Eye movements and cerebellar examinations were normal (Video 1).

**VIDEO 1:** Manifestations of parkinsonism and dystonia in the initial examination. Video content can be viewed at https://onlinelibrary.wiley.com/doi/10.1002/ccr3.72654.

## 3 Methods (Differential Diagnosis, Investigations, and Treatment)

**FIGURE 1:**
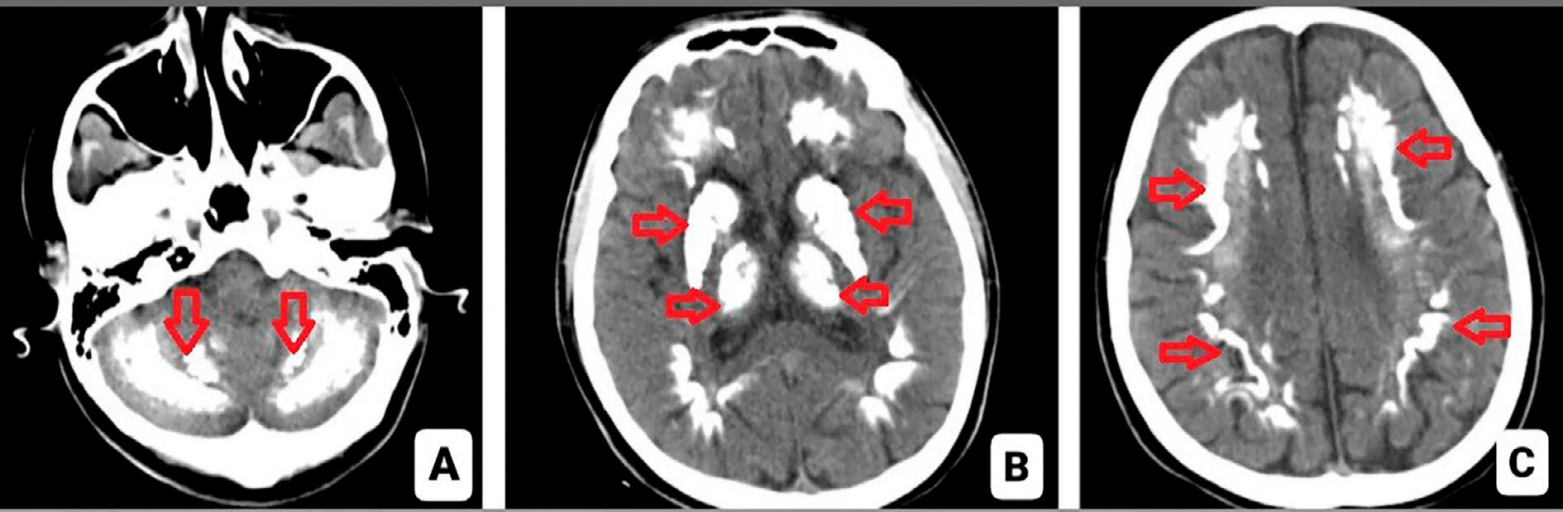
The red arrows show multiple bilateral calcifications in the cerebellar hemispheres (A), basal ganglia (B) and white matter (C).

In the brain CT scan, multiple bilateral calcifications were evident in the BG, white matter, and cerebellar hemispheres, and possible diagnoses of hypoparathyroidism, familial calcification, and Fahr syndrome were proposed for the patient (Figure 1).

The primary lab tests were all reported within the normal range (Table 1).

**TABLE 1:** The primary lab tests were all reported within the normal range.

Lab test	Serum level	Normal range
Sodium (Na)	135	135–145 mEq/L
Potassium (K)	3.7	3.5–5.5 mEq/L
Calcium (Ca)	8.4	8.5–10.5 mEq/L
Phosphorus (P)	3.7	3.4–4.5 mg/dL
Intact parathyroid hormone (iPTH)	27	10–55 pg/mL
Ceruloplasmin	26	20–35 mg/dL

In this patient, due to the normal serum calcium and iPTH levels, metabolic disorders such as hyperparathyroidism are less likely to occur. Also, due to the normal serum ceruloplasmin level and the absence of a KF ring in the eye examination, the diagnosis of Wilson's disease is ruled out. On the other hand, due to the hyperdensity in the CT scan and the lack of any similar family history, the diagnosis of neurodegeneration with brain iron accumulation (NBIA) cannot be considered for the patient.

According to the history and the findings of the paraclinical evaluations and lab tests, Fahr syndrome was suggested for the patient. Amantadine 100 mg, two tablets per day, levodopa/carbidopa 100/25 twice a day, and sertraline 50 mg daily were started.

## 4 Conclusion and Results (Outcome and Follow‐Up)

After 3 months of follow‐up, there was a slight improvement in parkinsonism and dystonic postures. This case report illustrates that recognizing psychiatric and cognitive symptoms, as well as parkinsonism and dystonia, coupled with distinctive imaging results, was critical in establishing the underlying illness. This report, along with findings of imaging and symptom presentation, can be helpful to increase understanding of this disease because our current knowledge of fahr syndrome is limited and the set of symptoms in these patients is diverse including psychiatric symptoms, behavioral and cognitive changes, and abnormal movements.

## 5 Discussion

It is essential to emphasize that distinguishing between Fahr's disease and Fahr's syndrome is crucial, in which there are specific causes for the calcification of the basal ganglia, such as hypoparathyroidism. Senile calcification of the basal ganglia without clinical symptoms with no underlying cause is an incidental finding that is seen in some people over 60 years old. These are also important differentials [6]. When psychiatric symptoms and abnormal movements coincide with idiopathic calcification of the brain in a patient, Fahr syndrome is suggested, especially if it is accompanied by neurological symptoms, cognitive deficits, and some degree of mental retardation [7].

The gradual onset of symptoms in this patient characterized by behavioral changes and a lower‐than‐normal IQ has been reported in several studies [8, 9]. Psychiatric manifesting in the patient's third decade of life, followed by movement disorders, is consistent with the early‐onset variant of Fahr's disease [10].

Despite the co‐occurrence of behavioral changes and neurological symptoms, along with neuroimaging findings that suggest Fahr syndrome, it has been reported that there is no clear connection between calcifications and neurological symptoms [11]. The treatment goals involve providing symptomatic support. The response to levodopa in those with Parkinsonian features is reportedly poor. Atypical antipsychotics are the preferred choice for psychiatric symptoms due to the coexistence of the extrapyramidal syndrome in this group of patients [12].

## Author Contributions

**Mohammad Javad Sinaeefar:** data curation, investigation, writing – original draft, writing – review and editing. **Tara Khoeini:** methodology, software, validation, writing – original draft, writing – review and editing.

## Funding

The authors have nothing to report.

## Ethics Statement

The researchers were committed and adhered to the principles of the Helsinki Convention and the Ethics Committee of the Iran University of Medical Sciences in all stages.

## Consent

After providing the necessary explanations, written informed consent was obtained from the patient regarding the submission of their clinical condition to medical journals. Additionally, the patient has been assured that their name and personal details will be kept confidential by the authors.

## Data Availability

The data that support the findings of this study are available from the corresponding author upon reasonable request.

## References

[1] F. Amisha and S. Munakomi , Fahr Syndrome. StatPearls [Internet], (StatPearls Publishing, 2023).

[2] S. Katwal , S. Bhandari , A. Ghimire , and P. Ghimire , “Fahr's Syndrome With Hypoparathyroidism, Thrombocytopenia, and Seizure: A Rare Case Report,” Annals of Medicine and Surgery 85, no. 8 (2023): 4131–4133. 37554906 10.1097/MS9.0000000000001032 PMC10406094

[3] J. P. Rissardo , A. L. F. Caprara , and J. O. F. Silveira , “Fahr's Disease Presenting With Pure Dementia: A Case Report and Literature Review,” Apollo Medicine 16, no. 4 (2019): 236–239.

[4] A. Balck , S. Schaake , N. S. Kuhnke , et al., “Genotype–Phenotype Relations in Primary Familial Brain Calcification: Systematic MDSGene Review,” Movement Disorders 36, no. 11 (2021): 2468–2480. 34432325 10.1002/mds.28753

[5] M. Carecchio , M. Mainardi , and G. Bonato , “The Clinical and Genetic Spectrum of Primary Familial Brain Calcification,” Journal of Neurology 270, no. 6 (2023): 3270–3277. 36862146 10.1007/s00415-023-11650-0 PMC10188400

[6] M. L. Perugula and S. Lippmann , “Fahr's Disease or Fahr's Syndrome?,” Innovations in Clinical Neuroscience 13 (2016): 45–46. 27672489 PMC5022990

[7] M. Rücker , W. Halder , M. Kofler , R. Ehling , and C. Brenneis , “Levodopa‐Responsive Dystonia and Parkinsonism in Fahr Syndrome,” Neurology: Clinical Practice 9, no. 6 (2019): 527–529. 32042500 10.1212/CPJ.0000000000000663 PMC6927444

[8] S. Naqvi , S. Arshad , R. Hanif , and K. A. H. Elfert , “Fahr's Syndrome Misdiagnosed as Schizophrenia: A Case Report,” Cureus 9, no. 3 (2017): e1071. 28473946 10.7759/cureus.1071 PMC5413360

[9] G. Palu , S. T. Moraes , G. Romaniello , et al., “Could Fahr's Syndrome Have More Than One Simultaneous Etiology?,” Cureus 13, no. 12 (2021): e20342. 35036185 10.7759/cureus.20342 PMC8752340

[10] K. Aghemo , R. Salmanzadeh , O. DeAngelo , and A. M. Salmanzadeh , “Advanced Early‐Onset Fahr's Disease: A Case Report,” Cureus 15, no. 5 (2023): e39495. 37362501 10.7759/cureus.39495 PMC10290546

[11] M. Pistacchi , M. Gioulis , F. Sanson , and S. Z. Marsala , “Fahr's Syndrome and Clinical Correlation: A Case Series and Literature Review,” Folia Neuropathologica 54, no. 3 (2016): 282–294. 27764521 10.5114/fn.2016.62538

[12] A. G. Asokan , S. D'souza , J. Jeganathan , and S. Pai , “Fahr's Syndrome‐An Interesting Case Presentation,” Journal of Clinical and Diagnostic Research 7, no. 3 (2013): 532–533. 23634413 10.7860/JCDR/2013/4946.2814 PMC3616573

